# Topographical study of the trapezius muscle, greater occipital nerve, and occipital artery for facilitating blockade of the greater occipital nerve

**DOI:** 10.1371/journal.pone.0202448

**Published:** 2018-08-15

**Authors:** Hyung-Jin Won, Hyun-Ju Ji, Jae Kyeong Song, Yeon-Dong Kim, Hyung-Sun Won

**Affiliations:** 1 Department of Anatomy, School of Medicine, Kangwon National University, Chuncheon, Korea; 2 Department of Mortuary Science, Eulji University, Seongnam, Korea; 3 Surgical Anatomy Education Center, Yonsei University College of Medicine, Seoul, Korea; 4 Department of Anesthesiology and Pain Medicine, Wonkwang Institute of Science, Wonkwang University College of Medicine, Iksan, Korea; 5 Department of Anatomy, Wonkwang University College of Medicine, Iksan, Korea; Johns Hopkins School of Medicine, UNITED STATES

## Abstract

The aim of this study was to clarify the topographical relationships between the greater occipital nerve and the trapezius muscle and between the greater occipital nerve and the occipital artery in the occiput in order to increase the success rate of greater occipital nerve blockade. Fifty-six halved heads of 28 cadavers were used in this study. The piercing points and the courses of the greater occipital nerve and occipital artery were analyzed by dividing a line connecting between the external occipital protuberance and mastoid process into three equal parts. A circle with a radius of 2 cm drawn at the medial trisection point of this line was divided into four equal sectors. The greater occipital nerve simply passed the lateral border of the trapezius muscle and then pierced the fascia connecting the cranial attachment of the trapezius muscle with the sternocleidomastoid muscle in 62.5% of the specimens, whereas it pierced the muscle itself in the other 37.5%. The greater occipital nerve and occipital artery pierced the fascia within the 2-cm-radius circle in 85.7% and 98.2% of the specimens, respectively. The piercing points of the greater occipital nerve and occipital artery were observed most frequently in the inferomedial (42.9%) and inferolateral (37.5%) sectors of the circle, respectively. The greater occipital nerve and occipital artery pierced the same sector of the circle and accompanied each other in 51.8% of the specimens. These results are expected to improve the understanding of the topographical relationships between the greater occipital nerve and trapezius muscle and between the greater occipital nerve and occipital artery in the occiput, and thus provide helpful information for the management of occipital neuralgia.

## Introduction

Occipital neuralgia manifests as paroxysmal stabbing pain in the skin areas corresponding to the passages of the greater occipital nerve (GON) and lesser occipital nerve [1], and can be considered one of the causes of cervicogenic headache. The prevalence of cervicogenic headache has been estimated at 15–20% in patients with chronic headaches [2]. Anatomical recognitions of the greater, lesser, and third occipital nerves with different patterns of pain, neurologic origins, and treatment modalities have been facilitated by the use of precision diagnostic injections and the search for more-aggressive methods for treating intractable headaches and upper cervical neck pain.

The GON presents variable courses in the occiput, and some of them have been thought to affect the entrapment neuropathy of this nerve related to cervicogenic headache. GON blockade has been reported to be one of the most useful modalities for managing cervicogenic headaches [3], [4], [5]. Many anatomical studies have been performed to identify the locations of the GON based on various landmarks such as the external occipital protuberance, superior nuchal line, and certain lines connecting between the external occipital protuberance and the mastoid process or auricle in order to determine the optimum injection points for performing GON blockade [6], [7], [8], [9], [10], [11]. The external occipital protuberance and occipital artery (OA) have been shown to be the most useful landmarks for detecting the GON around the superior nuchal line during occipital nerve injections [5].

This study was designed to clarify the topographical relationships between the GON and the trapezius muscle and between the GON and OA in the occiput, with the aim of facilitating the diagnosis and treatment of GON entrapment neuropathy.

## Materials and methods

Fifty-six halved heads of 28 embalmed Korean cadavers (15 males, 13 females; mean age at death, 64.5 years; age range at death, 31–86 years) were used for this study. None of the cadavers exhibited any evidence of gross pathology, previous surgical procedures, or traumatic lesions to the occiput. All cadavers stored in the Jesaeng-Euisae Institute of Wonkwang University College of Medicine qualified as materials for use in education and research according to the domestic law. This study was approved by the institutional review board of the Wonkwang University (IRB ID No. WKIRB-201708-BR-065).

The subcutaneous tissue layer was exposed after removing the skin of the occiput. The GON and OA were then carefully dissected without changing their locations in the occiput. All distances were measured with the aid of digital calipers (Mitutoyo, Tokyo, Japan). Except where stated otherwise, the data are presented as mean±SD values.

**Fig 1:**
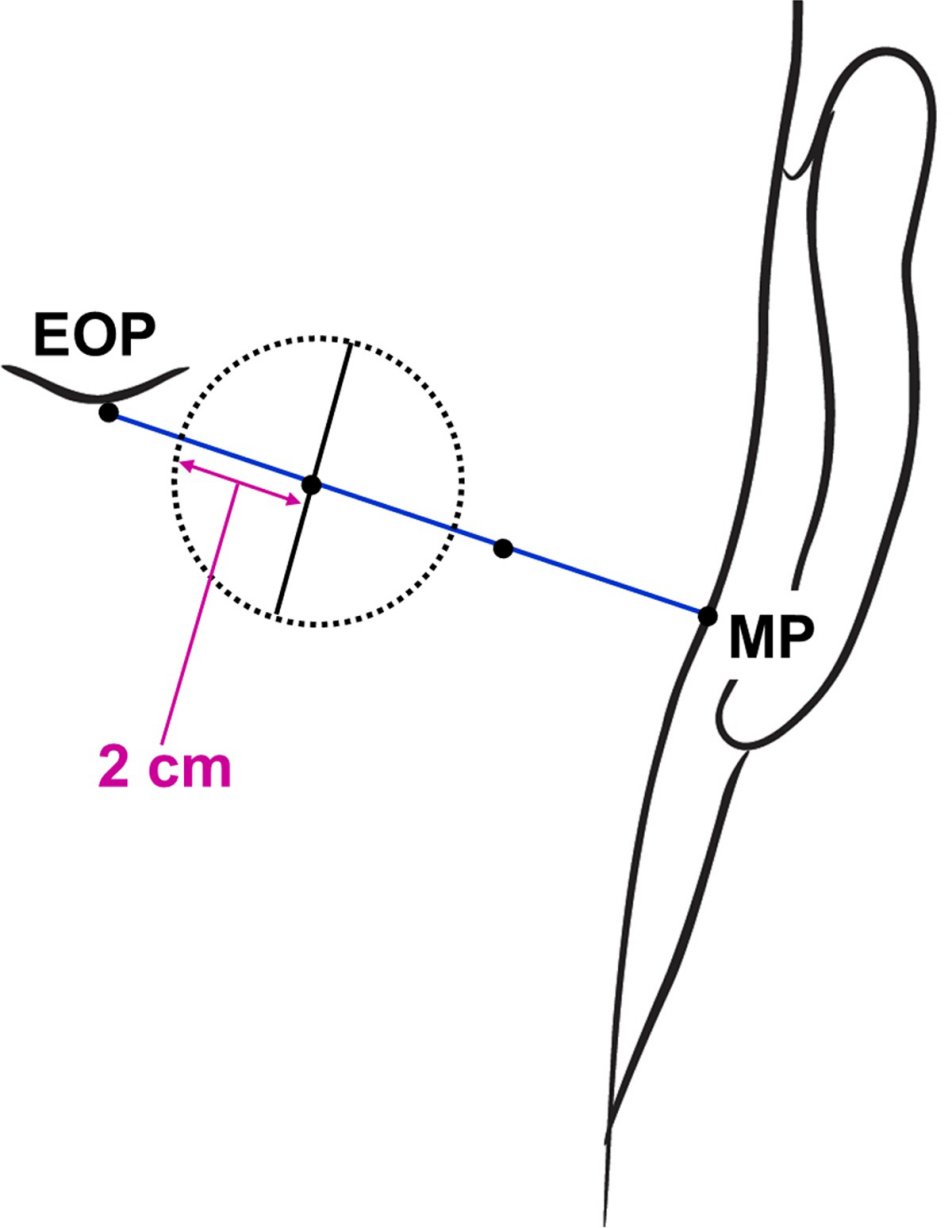
Circle defined for analyzing the locations of the GON and OA. EOP, external occipital protuberance; MP, mastoid process.

The authors defined a circle for analyzing the locations at which the GON and OA pierced the trapezius muscle or the fascia and the courses of the GON and OA after piercing these structures. First, a line connecting between the external occipital protuberance and the mastoid process was divided into three equal parts. A circle with a radius of 2 cm drawn at the medial trisection point of this line was divided into four equal sectors: superomedial, superolateral, inferomedial, and inferolateral (Fig 1).

## Results

**Fig 2:**
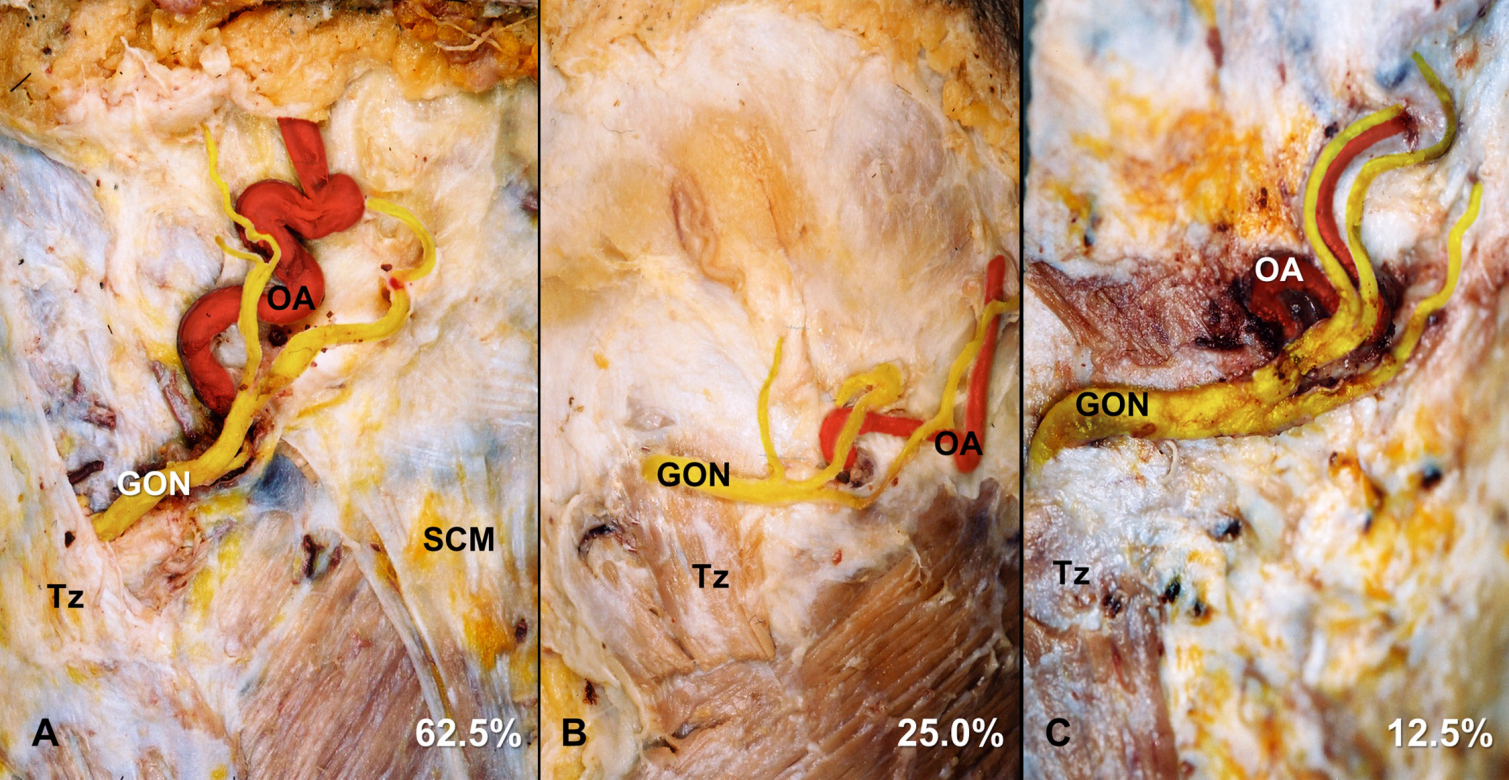
Different anatomical relationships between the GON and trapezius muscle at the superior nuchal line: (A) the GON simply passes the lateral border of the trapezius muscle comprising the aponeurosis, or the GON pierces the flesh (B) or aponeurotic (C) parts of the trapezius muscle. The lower parts of the trapezius muscles were cut and removed in panels B and C. Tz, trapezius muscle; SCM, sternocleidomastoid muscle.

The GON simply passed the lateral border of the trapezius muscle and then pierced the fascia connecting the cranial attachment of the trapezius muscle with the sternocleidomastoid muscle in 62.5% of the specimens (Fig 2A), whereas it pierced the muscle itself in the other 37.5% (Fig 2B and 2C). The GON was observed as a single trunk before piercing the fascia or the muscle in 83.9% of the specimens (Fig 2). The GON passed the lateral border of the trapezius muscle in its flesh part in 32.1% of the specimens and in its aponeurotic part in 30.4% of them. The GON pierced the trapezius muscle in its flesh and aponeurotic parts in 25.0% (Fig 2B) and 12.5% (Fig 2C) of the specimens, respectively. The GON pierced the muscle and fascia at 18.9±9.4 mm (range, 0.5–45 mm) vertically below the external occipital protuberance and 22.7±7.7 mm (range, 4–39 mm) lateral to the midsagittal line.

The OA pierced the fascia connecting the cranial attachment of the trapezius muscle with the sternocleidomastoid muscle and then ascended tortuously in the subcutaneous tissue layer of the scalp (Fig 2). The OA pierced the fascia at 17.0±9.2 mm vertically below the external occipital protuberance and 33.7±9.9 mm lateral to the midsagittal line, which was lateral to that of the GON in 85.7% of the specimens.

The GON and OA pierced the fascia within the circle in 85.7% and 98.2% of the specimens, respectively. The piercing point of the GON was observed most frequently in the inferomedial sector of the circle (42.9% of the specimens), followed by in the inferolateral (17.9%), superomedial (14.3%), and superolateral (10.7%) sectors. The piercing point of the OA was found most frequently in the inferolateral sector of the circle (37.5% of the specimens), followed by in the superolateral (33.9%), inferomedial (16.1%), and superomedial (10.7%) sectors.

**Fig 3:**
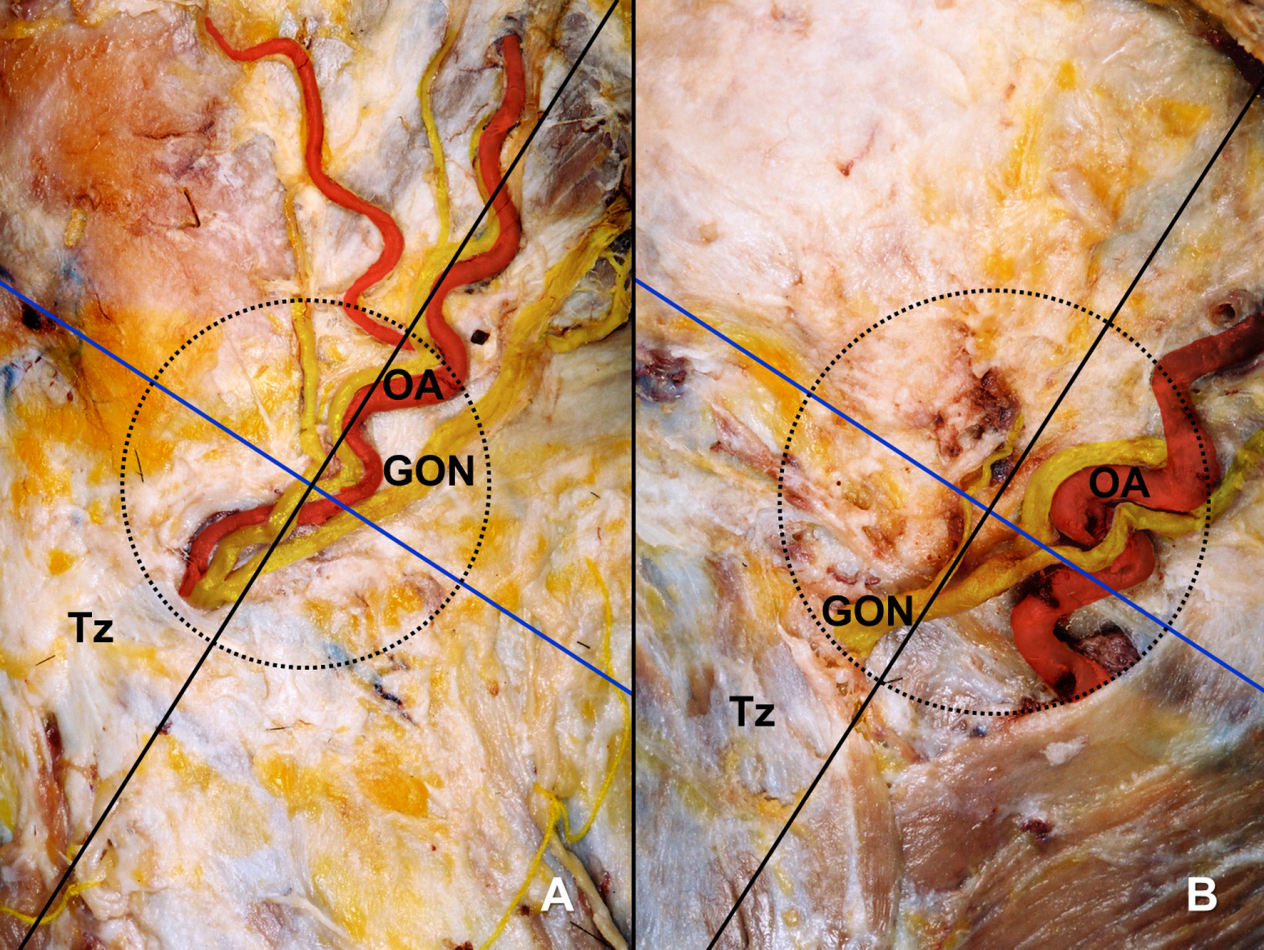
The GON and OA within a circle of radius 2 cm drawn at the medial trisection point of a line connecting between the external occipital protuberance and mastoid process. (A) The piercing points of the GON and OA are both located within the inferomedial sector of the circle. (B) The piercing points of the GON and OA are located within the inferomedial and inferolateral sectors of the circle, respectively. Tz, trapezius muscle.

**Fig 4:**
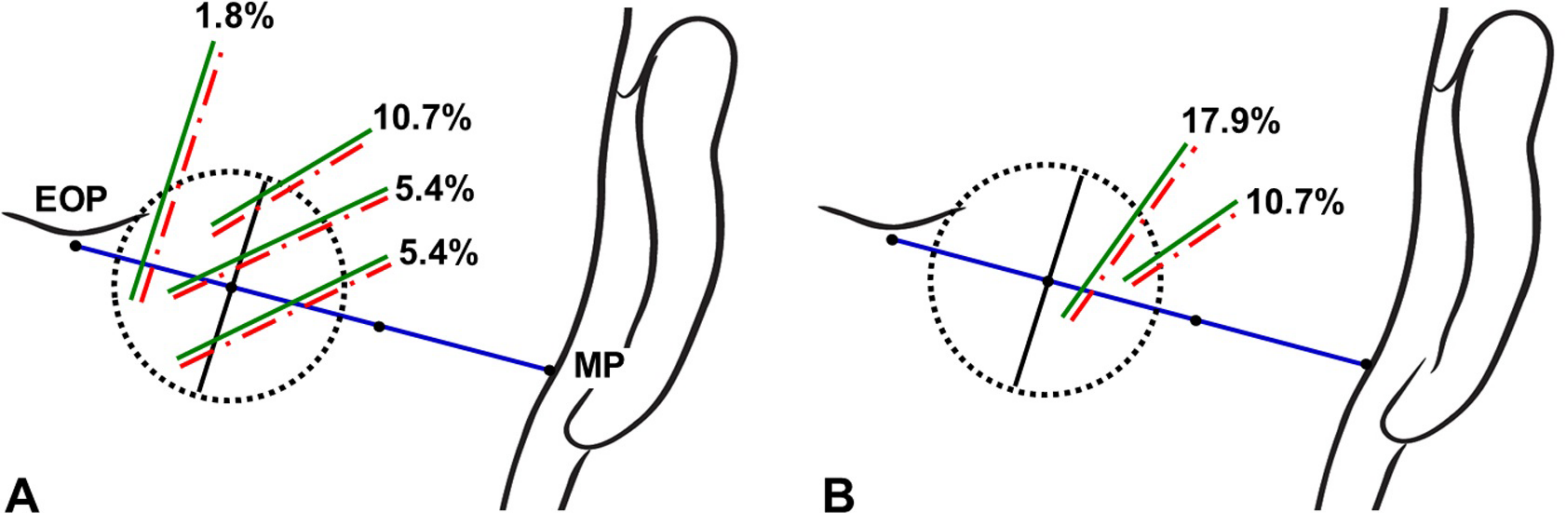
Incidence rates of the GON and OA piercing both the same sectors of the circle. The green and red dotted-dashed lines indicate the GON and OA, respectively.

**Fig 5:**
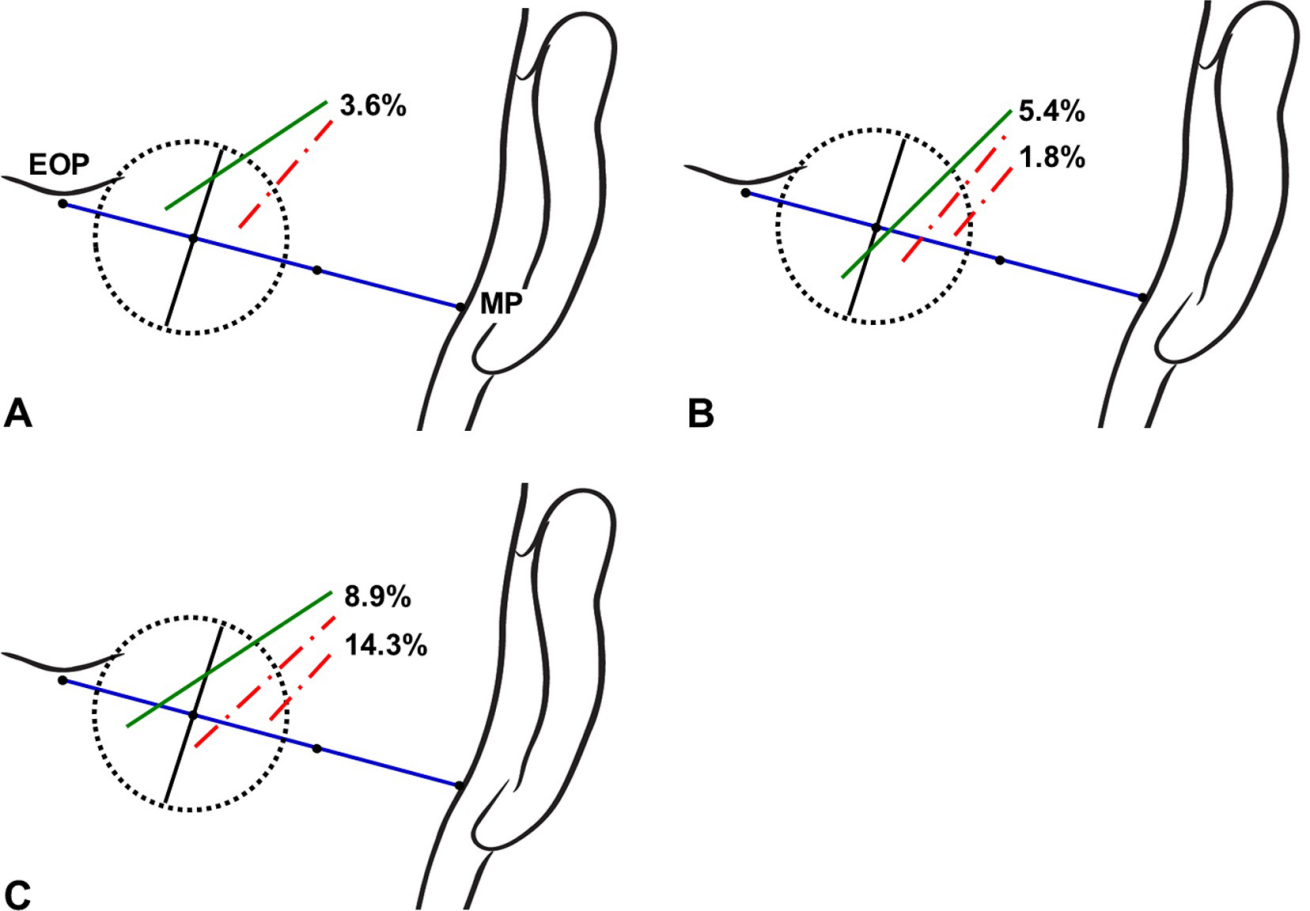
Incidence rates of the GON and OA piercing each different sectors of the circle. The green and red lines indicate the GON and OA, respectively.

**Fig 6:**
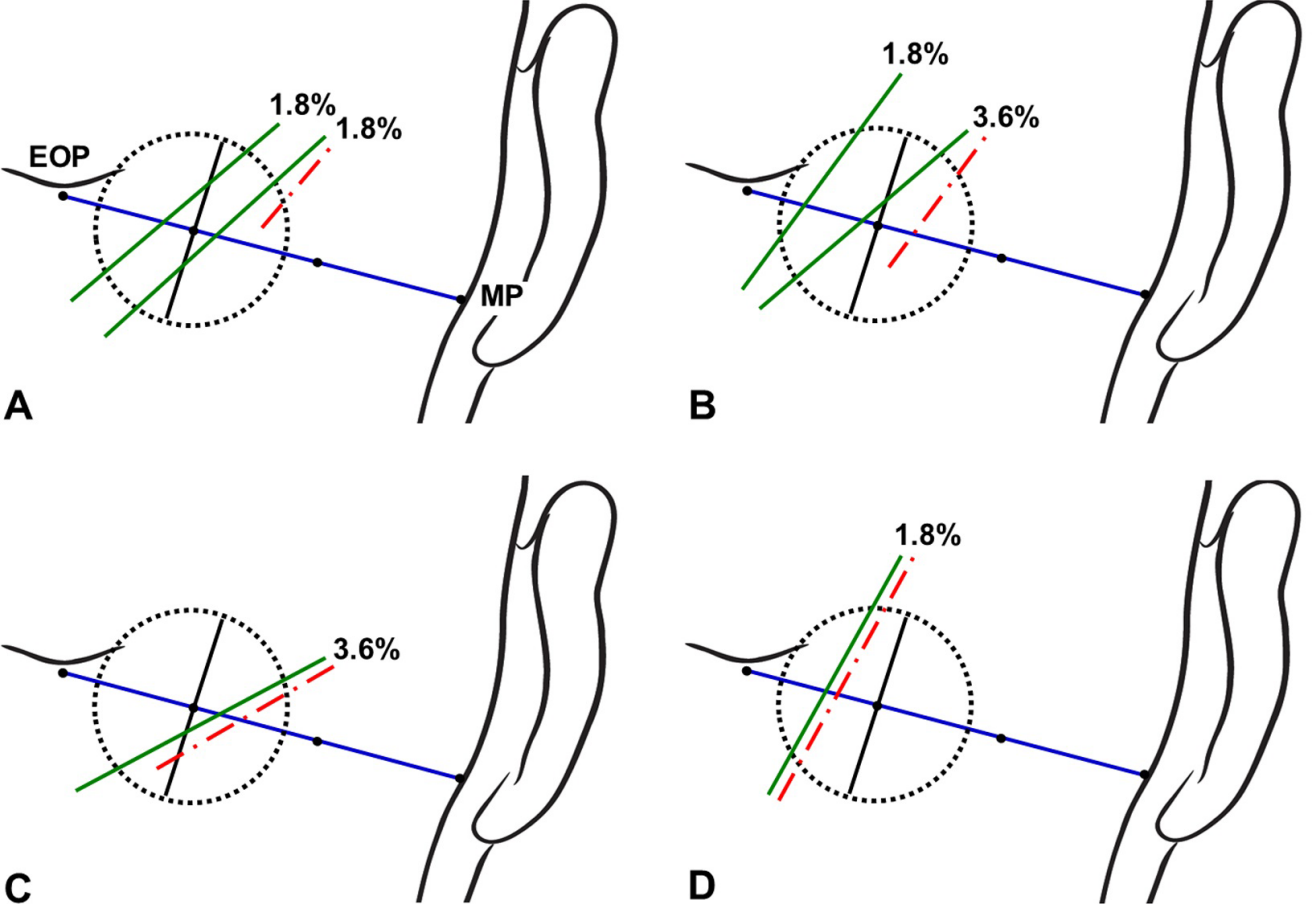
Incidence rates of the GON or both the GON and OA pierced the fascia outside the circle. The green and red lines indicate the GON and OA, respectively.

The GON and OA pierced the same sector of the circle and accompanied each other in 51.8% of the specimens (Figs 3A and 4). In 33.9% of the specimens, the GON and OA each pierced different sectors of the circle (Figs 3B and 5). In another 14.3% of the specimens, the GON (12.5%) or both the GON and OA (1.8%) pierced the fascia in the area outside the circle (Fig 6).

## Discussion

Diagnostic injections for relieving the patient’s symptoms are the only reliable technique for diagnosing occipital neuralgia [12]. Various therapeutic occipital interventions are applied, including treatment using local anesthetic injection with steroid, occipital neurolysis, occipital neurectomy, radiofrequency denervation, and peripheral nerve stimulation for each affected occipital nerve [13], [14], [15], [16]. GON blockade has also been found to be effective for managing occipital neuralgia.

In the diagnosis and treatment of GON entrapment neuropathy, the understanding of the course of the nerve, especially the relationship between the nerve and adjacent muscle is essential. The GON arising from the medial branch of the C2 dorsal ramus traverses the obliquus capitis inferior muscle, and then pierces both of the semispinalis capitis and the trapezius muscles near their occipital attachments [17]. After piercing these muscles the GON travels with the OA. The point where the GON passes between the atlas and the axis [18], [19], or the point at which the nerve runs between the obliquus capitis inferior and semispinalis capitis muscles [7], or the point at which the nerve penetrates the semispinalis capitis muscle and the aponeurotic part of the trapezius muscle [7], has been reported as the major possible zones of GON irritation and entrapment. These points are highly likely to be affected by various factors such as whiplash injury resulting in spasm of the trapezius muscle, repetitive neck contractions resulting from the work environment, and an unbalanced posture related to the head-forward position. These mechanisms can all be causative origins of occipital neuralgia.

The descriptions of these possible zones related to the trapezius muscle and GON have differed among researchers. Bovim et al. [8] reported that the GON pierced the trapezius muscle in 45% of 40 cases, and coursed laterally to the insertion point without piercing the muscle in the other 55%. Tubbs et al. [20] found that the GON either pierced the trapezius muscle (in 16.7% of 20 cases) or its aponeurosis (83.3%). Bogduck [6] reported that the GON did not penetrate the trapezius muscle in all of five cases, instead emerging through an aperture above the aponeurotic sling between the trapezius and sternocleidomastoid muscles. In the present study, the GON pierced the flesh part (25% of 56 cases) or the aponeurotic part (12.5%) of the trapezius muscle, or simply passed the lateral border of the trapezius muscle (62.5%). These anatomical variations constitute valuable information when deciding the injection site in patients with myofascial pain syndrome related to the trigger point of the upper trapezius muscle that results in posttraumatic neck pain or headache [21].

Successfully identifying the location of the GON for both diagnostic and therapeutic purposes requires the identification of landmarks for determining injection sites accurately. In a blind technique, the injection point is recommended where the GON crosses the superior nuchal line approximately halfway or one third between the external occipital protuberance and the mastoid process [9], [22]. The OA emerges on this line as well as the GON, and is known to be typically lateral to the GON [22]. The OA has been known to be easily found by using finger and ultrasound regarded as fast, convenient, and inexpensive diagnostic device. This artery, thus, has been regarded as the most-reliable anatomical landmark when performing GON blockade [23], [24]. However, the OA has been reported that it sometimes cannot be detected by pulsation with the finger in a blind technique [25]. Moreover, the present study revealed that the OA did not run lateral to the GON in 14.3% of cases (Fig 3A), which means that false positive or negative response might be happen in some patients even after GON blockade with ultrasound [26].

The injection point for performing GON blockade based on using bony landmarks has been inconsistent among previous studies. Bovim et al. [8] blocked the GON at a point about 2 cm lateral to and 2 cm below the external occipital protuberance. Ashkenazi and Levin [27] anesthetized the GON at 3.5 cm inferolaterally to the external occipital protuberance. Natsis et al. [10] recommended injecting patients at 2.0‒2.5 cm inferior to the external occipital protuberance and approximately 1.5 cm lateral to the midline. In the present study, the GON was found to pierce the fascia connecting the cranial attachment of the trapezius muscle with the sternocleidomastoid muscle or the trapezius muscle itself at about 1.9 cm vertically below the external occipital protuberance and about 2.3 cm lateral to the midsagittal line. The circle defined in the present study could cover almost the entire area where the GON coincides with the conventional landmarks, but the piercing point of the GON appearing outside the circle (which occurred in 14.3% of the specimens) should be considered as one cause of refractoriness to GON blockade (Fig 6).

**Fig 7:**
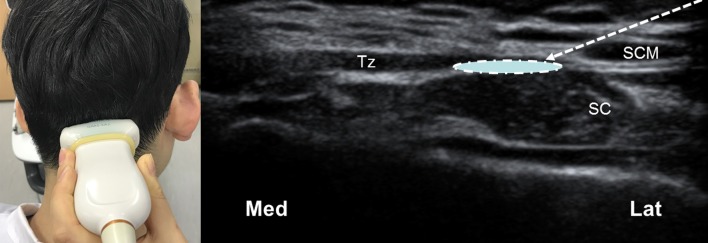
Another suggested injection point for GON blockade with ultrasound guidance. The linear transducer (L12-5 MHz, HDI 5000, Philips, USA) is positioned to allow a short-axis view. The arrow and circle indicate the needle trajectory and the target point for spreading local anasthetics, respectively. Med, medial side; Lat, lateral side; SC, semispinalis capitis muscle.

In conclusion, the failure to obtain a blockade effect could be due to anatomical variations, and so topographical relationships between the GON and the trapezius muscle or the OA should be reconsidered in cases of refractoriness to injection therapy. The present results also indicate that when using ultrasound, another suitable injection site could be considered where the GON passes the lateral border of the trapezius muscle (Fig 7). It should be noted additionally that when using either a blind or ultrasound-guided technique, conventional landmarks do not always cover most of the GON and OA, which can also result in unsuccessful GON blockade. However, this study has the limitation that it could not suggest the accurate success rate or optimal quantity of local anesthetics for GON blockade in the various region, because this was an observational study performed using a limited number of fixed cadavers. It should be resolved in future studies such as a cadaveric study using dye injection or clinical trial in living populations based on the result of our study.

## Supporting information

S1 File A file (XLSX) containing raw data of study subjects. (XLSX)
